# The use of biomarkers associated with leaky gut as a diagnostic tool for early intervention in autism spectrum disorder: a systematic review

**DOI:** 10.1186/s13099-021-00448-y

**Published:** 2021-09-13

**Authors:** Laila Al-Ayadhi, Naima Zayed, Ramesa Shafi Bhat, Nadine M. S. Moubayed, May N. Al-Muammar, Afaf El-Ansary

**Affiliations:** 1 Department of Physiology, Faculty of Medicine, King Saud University, Riyadh, Saudi Arabia; 2 Autism Research and Treatment Center, Riyadh, Saudi Arabia; 3 Therapuetic Chemistry Department, National Research Centre, Dokki, Cairo, Egypt; 4 Biochemistry Department, College of Sciences, King Saud University, Riyadh, Saudi Arabia; 5 Botany and Microbiology Department, College of Science, Female Campus, King Saud University, Riyadh, Saudi Arabia; 6 Department of Community Health, College of Applied Medical Sciences, King Saud University, Riyadh, Saudi Arabia; 7 Central Laboratory, Female Centre for Scientific and Medical Studies, King Saud University, P.O box 22452, Zip code 11495 Riyadh, Saudi Arabia

**Keywords:** Autism, Leaky gut, Gut microbiota, Short-chain fatty acids, Diet, Probiotics, Zonulin

## Abstract

**Background:**

Innovative research highlighted the probable connection between autism spectrum disorder (ASD) and gut microbiota as many autistic individuals have gastrointestinal problems as co-morbidities. This review emphasizes the role of altered gut microbiota observed frequently in autistic patients, and the mechanisms through which such alterations may trigger leaky gut.

**Main body:**

Different bacterial metabolite levels in the blood and urine of autistic children, such as short-chain fatty acids, lipopolysaccharides, beta-cresol, and bacterial toxins, were reviewed. Moreover, the importance of selected proteins, among which are calprotectin, zonulin, and lysozyme, were discussed as biomarkers for the early detection of leaky gut as an etiological mechanism of ASD through the less integrative gut–blood–brain barriers. Disrupted gut–blood–brain barriers can explain the leakage of bacterial metabolites in these patients.

**Conclusion:**

Although the cause-to-effect relationship between ASD and altered gut microbiota is not yet well understood, this review shows that with the consumption of specific diets, definite probiotics may represent a noninvasive tool to reestablish healthy gut microbiota and stimulate gut health. The diagnostic and therapeutic value of intestinal proteins and bacterial-derived compounds as new possible biomarkers, as well as potential therapeutic targets, are discussed.

**Supplementary Information:**

The online version contains supplementary material available at 10.1186/s13099-021-00448-y.

## Background

The concept of the “leaky gut” has received increasing attention in the scientific community because of its association with several gastrointestinal (GI) and non-GI diseases, such as irritable bowel syndrome, most neurological disorders, asthma, type 2 diabetes, and many others [1], and also because of the fact that dietary modifications, probiotics, and other interventions are proposed to increase “gut integrity” as a treatment strategy for multiple human diseases [2, 3]. An understanding of the human gut microbiota in terms of lining the intestinal lumen, composition, and function has increased recently, largely because new technologies have revealed the genetic and metabolic profiles of the gut microbial strains as a new organ in the body, with a new promising therapeutic route for many disorders. It is more likely to compare it to a group of cells working in unity with the host, sometimes promoting health and other times leading to disease. It is crucial to understand the role of physical barriers, gut microbiota diversity, and the effects of different diets in inducing leaky gut as an important etiological mechanism in GI problems, which are co-morbidities in autism spectrum disorder (ASD).

About 400 m^2^ of the surface area in humans is covered by the intestinal mucosal barrier, which serves as a physical barrier [4]. It is made up of a thin layer of epithelial cells, composed of multiple cell types. The enterocytes (intestinal epithelial cells [IECs]) are the most abundant cell type, forming an operational barrier to protect the internal milieu. In addition to its protective function, enterocytes control the selective absorption of useful ions, nutrients, and other constituents from the lumen to the blood. Among the enterocytes, goblet cells are in charge of the secretion of mucus, and enteroendocrine cells produce intestinal hormones, peptides, and neurotransmitters. Antimicrobial compounds that are important in immunity and host defense are produced by Paneth cells, and intestinal tolerance is usually controlled by M cells and goblet cells [4]. Moreover, Bohorquez et al.[5] supports a conserved relationship between enteroendocrine cells and enteric glial cells as essential cells for the normal development of enteric neurons and for the maintenance of the intestinal epithelium integrity. The transport between these IECs and the intestinal epithelium, known as the paracellular pathway, is controlled by various tight junction (TJ) proteins [6]. TJs are semipermeable intercellular adhesion complexes in epithelia and endothelia that regulate intestinal permeability. These junctions cross-talk through direct protein–protein interactions, as well as by transmitting signals to each other that influence their assembly and function. Ion permeability at TJs is determined largely by a family of transmembrane proteins, known as claudins that are thought to form gated ion-selective paracellular pores through the diffusion barrier. They form bidirectional signaling boards that transduce signals to the cell interior to control cell differentiation and survival, and receive signals from the interior of the cell that control their assembly and function [6].

Additionally, commensal bacteria forming the gut normal flora (gut microbiota) are considered a major component of the physical barrier [7] because of their role in preventing the colonization of pathogenic bacteria, releasing antimicrobial metabolites, competing for nutrients, and providing sites for attachment [8, 9]. Together, the IEC and the gut microbiota regulate the physical barrier by inhibiting the entrance of harmful substances; consequently, any disturbance due to various pathological conditions lead to impaired gut homeostasis [10] and impaired gut permeability, known as “leaky gut.”

Bearing in mind the complexity and challenges to characterize the gut microbiota, it is often seen as a two-phylum system (the Firmicutes and Bacteroidetes) [11], present mainly in the GI tract in different ratios, depending on the individual itself and many other factors, including diet, diseases, and stress. Currently, we do not know how this ratio varies; however, the relative abundance of a group of organisms translates the alteration in the gut microbiota composition. There is evidence that depletion of a single species can cause deficiency dysbiosis and leaky gut, leading to other associated disorders [12].

Differences in a strain within a species can also cause a variation to be either pathogenic or beneficial; for instance, *Escherichia coli* could be used as a probiotic, although in other cases, it is associated with inflammatory bowel disease (IBD) and colorectal cancer (CRC) [13, 14]. Although the mechanism of action of this microbial community toward the host is still unclear, its function and relationship with the host are vital. In fact, various species are being studied in the large intestine of different individuals and little is shared between these individuals. Surprisingly, these species perform the same functions in every individual’s GI tract. Thus, we can conclude that function is more important than the types of species present in the gut. However, differences in the gut microbial composition may lead to variations in the effectiveness of the function itself. For instance, obligate anaerobes (Firmicutes and Bacteroidetes) produce enzymes responsible for the complex hydrolysis of carbohydrates (resistant starch and fiber) that are nondigestible by the human host. *Lactobacillus* and *Bifidobacterium*, on the other hand, are two genera that synthesize short-chain fatty acids (SCFAs) called acetate, propionate, and butyrate [15]. SCFAs, through the interaction with innate pattern recognition receptors in the gut mucosa, can drive the steady-state expression of mucus and other antimicrobial elements. Despite the beneficial effects of low concentrations of these SCFAs, much higher concentrations could demonstrate an opposite effect, even inducing apoptosis in a concentration-dependent manner, leading to a disturbed homeostasis of the intestinal barrier [15]. Disruption of gut barrier homeostasis can lead to amplified inflammatory signaling, increased epithelial permeability, and dysbiosis of the microbiota. Additionally, gut–brain signaling may be affected by prolonged mucosal immune activation [15].

Furthermore, increased levels of interleukin-6, pro-inflammatory cytokines, and plasma lipopolysaccharide (LPS), in addition to physiological stress, can lead to small intestinal permeability known as “leaky gut,” allowing toxins and antigens produced by harmful bacteria to cross the intestinal lumen and pass into the bloodstream. Thus, maintaining normal gut flora is critical for building an effective intestinal barrier. Recent studies have reported that probiotic bacteria, such as *Bifidobacterium* and *Lactobacillus*, can enhance the production of TJ proteins, thus reversing the leaky gut disorders; however, additional and longer-term studies are still required. Certain probiotic species including, but not limited to, *Lactobacillus rhamnosus* [16–19]. *Streptococcus thermophilus* [20], *Bifidobacterium infantis* [21], and *Lactobacillus reuteri* [22] are considered as gatekeepers for the paracellular pathway, promoting healthy gut microbiota and a healthy gut–brain and gut–liver axis. Conversely, pathogenic bacteria that can facilitate a leaky gut and induce autoimmune symptoms can be ameliorated with the use of antibiotics. Clarifying the aspects that regulate the communication between microbes as regulators of intestinal barrier function and the immune system, and the gradual integration of this dialogue with the nervous and hormonal systems, is challenging as necessary steps for new emerging therapeutic strategies [23].

The gut–brain axis (GBA) includes highly interconnected body structures. Those systems are the central nervous system (CNS), the autonomic nervous system (vagal and spinal nerves), and the enteric nervous system (ENS). The latter consists of the arrangement of neurons and supporting cells along the GI tract. Other essential components of the GBA include the hypothalamic–pituitary–adrenal axis responsible for gut hormones, the immune system as a source of multiple cytokines, and bacteria-derived metabolites such as SCFAs and free amino acids. Failures in the GBA cross-talk may lead to a number of health disorders, from inflammatory to metabolic and neurodevelopmental conditions, including ASD [24].

The bidirectional cross-talk between the gut and the brain usually takes place through the production of neurotransmitters, triggering the release of gut hormones from entero-endocrine cells, stimulation of the ENS and signaling to the brain via ascending neural pathways, and activation of the immune system via cytokine release by the mucosa-associated immune cells [25].

## Autism and leaky gut

ASD are complex disorders associated with abnormal brain development, prominent to poor social interactions and communication, restricted interest, and repetitive behaviors [26]. In 2016, autism was reported to affect 1 in every 37 US children [27], with a significantly higher incidence in boys than in girls [28]. Individuals with ASD frequently have disorders of the gut, such as bloating, diarrhea, and constipation [29].

Recent studies report that altered gut microbiota could affect the brain functions and development through the GBA [29], which refers to the pathways of bidirectional interaction between the CNS and the trillions of microorganisms that reside in the gut. Nevertheless, the description of a distinctive ASD gut microbial pattern and its possible role in ASD remains undistinguishable [30]. Regardless of the fact that behavioral instabilities are not effective predictors of GI problems, as they are already common in ASD-affected children without GI problems [31], they could indicate an accepted association between GI dysfunction in ASD and abnormal autistic behavior through the GBA [32, 33]. Rosenfeld [34] suggested that if dysbiosis is revealed to be a triggering factor in ASD, then, several possible intervention approaches ranging from prebiotics, probiotics, symbiotics, fecal transplantation, to other strategies used to alter the microbiomes or products may be useful in treating these patients.

Actually, the presence of autistic phenotypes has been linked to a less diverse gut microbiota, with significantly less carbohydrate degrading and fermenting bacteria of the genera Prevotella, Coprococcus, and Veillonellaceae in autistic microflora samples than in the healthy controls [35]. Another study reported that *Clostridium* spp. and enterococci were isolated more frequently from stool samples of autistic children than from those of controls; significant differences were observed mainly among staphylococci, *Candida* spp., and *Clostridium perfringens*, and there was an increase in the Firmicutes/Bacteroidetes ratio [36]. Taken together, all these microbiome alterations could be associated with the increased GI disturbances in individuals with ASD. The variation of the microbial abundance in ASD patients could be attributed to multiple factors including genetic and environmental factors such as nutritional deficiencies or overloads, exposure to infection. Table 1 summarizes the abundance of bacterial species detected in ASD patients compared to matching healthy controls [36].

**Table 1:** Occurrence of bacterial genera in Autistic and Healthy individuals gut metagenome

Bacterial genera	Percentage of occurrence in Autistic individuals	Percentage of occurrence in Healthy individuals	Autistic/Healthy Ratio
*Akkermansia*	1.57 ± 2.26	0.81 ± 1.26	1.94
*Alistipes*	14.76 ± 13.51	13.18 ± 10.10	1.12
*Bacteroidales noname*	0.78 ± 1.20	1.14 ± 2.13	0.68
*Bacteroides*	43.97 ± 17.56	40.20 ± 17.55	1.09
*Barnesiella*	1.33 ± 2.83	2.60 ± 4.02	0.51
*Bifidobacterium*	1.20 ± 2.27	2.10 ± 3.70	0.57
*Blautia*	1.11 ± 1.48	0.96 ± 0.90	1.16
*Clostridium*	1.29 ± 5.26	0.35 ± 0.44	3.69
*Coprococcus*	0.59 ± 1.22	0.39 ± 0.93	1.51
*Dialister*	2.46 ± 6.62	2.33 ± 3.31	1.06
*Escherichia*	0.58 ± 1.39	0.35 ± 0.55	1.66
*Eubacterium*	3.97 ± 5.26	6.72 ± 8.17	0.59
*Faecalibacterium*	4.22 ± 4.30	5.40 ± 4.59	0.78
*Haemophilus*	0.80 ± 2.04	0.49 ± 0.73	1.63
*Lachnospiraceae noname*	1.58 ± 4.48	0.53 ± 0.71	2.98
*Odoribacter*	0.57 ± 0.67	1.07 ± 1.28	0.53
*Parabacteroides*	2.88 ± 2.49	4.48 ± 2.13	0.64
*Prevotella*	0.69 ± 2.04	1.03 ± 3.05	0.67
*Roseburia*	2.87 ± 3.09	1.92 ± 1.63	1.49
*Ruminococcus*	1.99 ± 2.87	3.38 ± 3.95	0.59
*Subdoligranulum*	2.20 ± 3.31	2.37 ± 3.53	0.93
*Sutterella*	0.59 ± 1.55	1.67 ± 3.83	0.35
*Veillonella*	1.67 ± 3.99	0.25 ± 0.34	6.68

Reused by permission from Microbiology Society, License ID: 1128650-1 on 25-june-2021

The increased production of LPS and pro-inflammatory cytokines by Gram-negative bacteria [37] has been suggested as contributing factors for the impairment of both the GBA and blood–brain barrier (BBB) found in patients with ASD [38]. Preclinical proof from germ-free (GF) mice proposes that the microbiota can modulate the BBB. The exposure of GF adult mice to the fecal transplant from pathogen-free mice reduced BBB permeability and increased the expression of TJ proteins [39]. Additionally, the monocolonization of the intestine of GF adult mice with SCFA-producing bacterial strains normalized BBB permeability, whereas sodium butyrate was associated with the increased expression of occludin in the frontal cortex and hippocampus [39]. This study strengthens the hypothesis that the BBB may also be vulnerable to changes in the gut microbiota, and suggests that gut microbiota–BBB cross-talk starts during pregnancy and propagates throughout life. Logsdon et al. [40] proved that bacterial factors can infiltrate the gut-associated lymphoid tissue (GALT) and the blood lumen, where they interact with various immune cells. Regulatory T-cells and altered gut microbiota can promote T-cell brain infiltration, upregulate inflammatory cytokine concentrations, and negatively affect BBB integrity, inducing neuroinflammation. LPSs from pathogenic bacteria can act on endothelial toll-like receptors and induce neuroinflammation. On the other hand, beneficial bacterial metabolites can upregulate TJ proteins and improve BBB integrity [39].

**Fig. 1:**
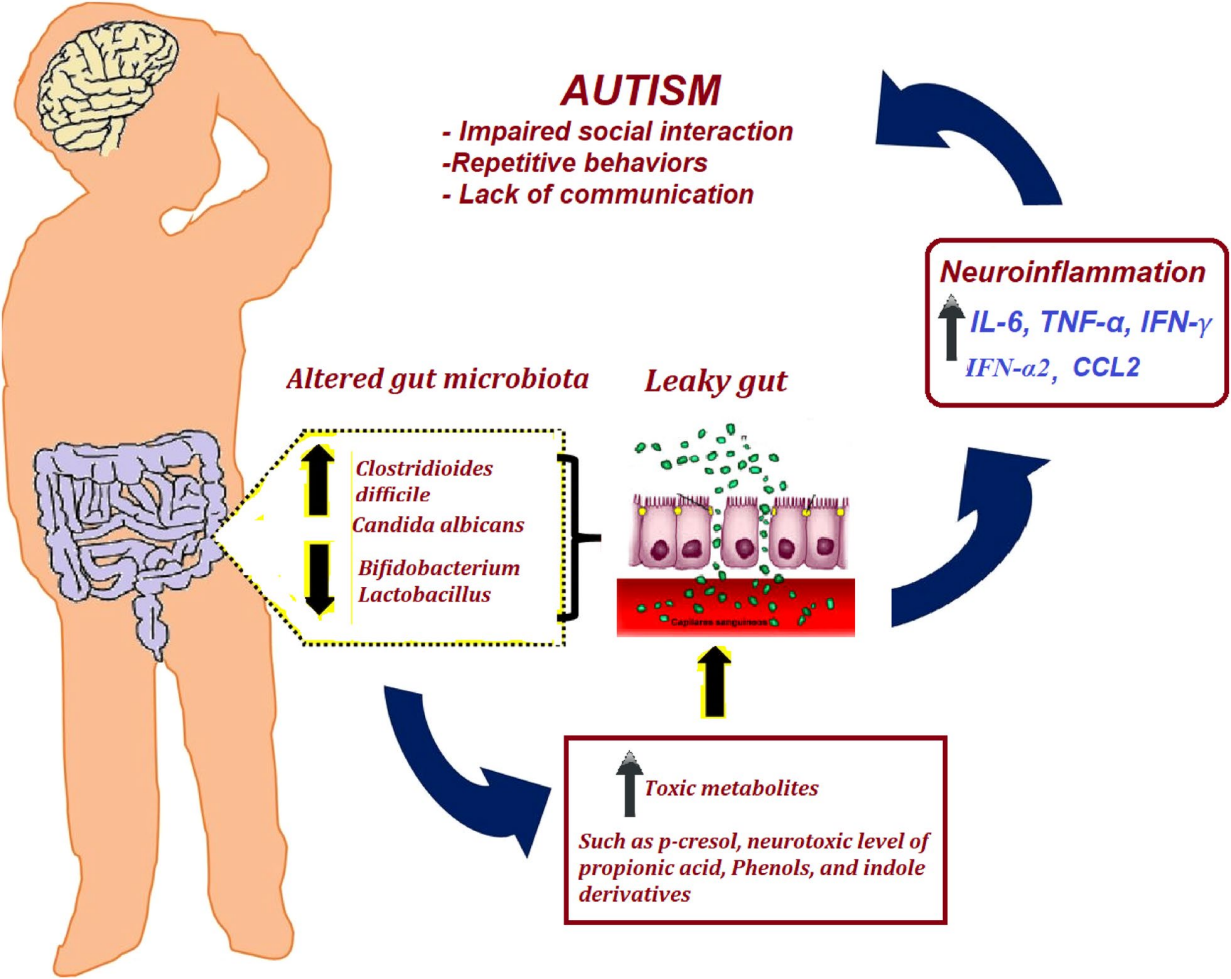
Role of impaired gut microbiota and leaky gut in the etiology of autistic features. Reduced abundance of probiotic bacteria together with the overgrowth of pathogenic bacteria and accumulation of their toxic metabolites are thought to induce leaky gut, release of pro-inflammatory cytokines, and the development of autistic features through the gut-brain axis

An increasing body of evidence indicates that children with ASD have altered gut microbiota [41]. A much higher concentration of *Candida albicans* was recorded in fecal samples of ASD-affected individuals than in those of normal healthy controls. Moreover, the presence of inflammatory markers was correlated with the severity of ASD phenotypes [42]. Luna et al. [43] compared the microbiome profile of ASD and neurotypical children with and without GI disorders using rectal biopsies. They recorded distinctive gut microbiota and inflammatory cytokines, such as CCL2 chemokine, eotaxin, and immune interferon-α2, in the rectal mucosa of ASD-affected children with GI disorders. Moreover, the plasma tryptophan level as a serotonin precursor was much higher in ASD-affected children with abdominal pain, which suggests the involvement of the serotonin pathway in the leaky gut as an autistic phenotype. Although normal gut microbiota provide a natural defense against pathogenic species through competition and maintenance of the mucosa, pathogenic bacterial species can promote a “leaky gut,” where metabolites associated with the microbes leave the gut and enter the bloodstream to reach the brain through the GBA [43]. Rose et al. [44] through comparisons of rectal and caecum biopsies between 10 ASD-affected children with GI complaints with those of 10 children with Crohn’s disease and 10 children with nonspecific GI complaints, showed that abnormalities in the mitochondrial activity of the gut mucosa are a contributing factor to GI problems and, hence, to the leaky gut of autistic patients. It is well known that dysbiosis is accompanied by a disrupted mucosal barrier that causes increased intestinal permeability of exogenous dietary peptides or neurotoxic bacterial peptides, such as LPSs, that leads to the production of inflammatory cytokines. This could suggest that the gut microbiota and related metabolites play a crucial role in the GBA, through which autistic phenotypes are induced [44] (Fig. 1)

## Biomarkers of leaky gut in ASD

Direct biomarkers of the gut microbiota, obtained through analyses of data from deep sequencing projects of biosamples from patients with neurological disorders, or indirectly through measurements of the toxic cycle of inflammation and gut permeability-related proteins in the blood, could help greatly in the diagnosis of ASD. As we progress in our understanding of the benefits and risks of certain combinations of microorganisms with regard to different neurological disorders and their clinical manifestations, it will be equally important to characterize host phenotypes that relate to specific microbial compositions. Biomarkers in blood, feces, or urine could be used to measure intestinal permeability in animals and humans. The permeability of the small intestine is assessed commonly by the measurement of intestinal permeation and urinary excretion of orally administered water-soluble, non-metabolizable sugars of different molecular weights. Usually, these analyses use large-size lactulose and small-size mannitol. Lactulose (L) is assumed to permeate paracellularly when the intestinal barrier is disrupted, whereas smaller molecules such as mannitol (M) are assumed to permeate both transcellularly and paracellularly, so the ratio of these two sugars (L/M) in plasma or urine reflects intestinal permeation, taking into account differences in the surface area of the epithelium [45]. D’Eufemia et al. [45] detected an increased L/M ratio in 9 of 21 (43%) autistic children, but in none of the healthy age-matched controls. The excreted mannitol fraction of the study’s autistic participants was similar to that of its control group, whereas lactulose excretion was greater than that in the control group. In another study, the increase in L/M values in both patients with ASD (36.7%) and their relatives (21.2%) was mainly due to the two- to threefold increase in lactulose compared with mannitol recovery [46].

Specifically, a leaky gut allows for the translocation of LPS, molecules found on the outer membrane of Gram-negative bacteria, from the gut into the circulation. LPS, in turn, activate various immune cells, leading to the increased secretion of pro-inflammatory cytokines and systemic low-grade inflammation. Compared with neurotypical healthy controls, serum levels of LPS were significantly higher in autistic individuals and inversely correlated with social interaction scores [47].

Lysozyme is a member of the alkaline glycosidases. It catalyzes Gram-negative bacterial cell wall reactions and protects the gut from pathogenic bacteria. The main source of fecal lysozyme is the intestinal granulocytes and mononuclear cells in the bowel’s lumen. Lysozyme is an important component of the immune system. Through the use of a cellular automaton model, the interdependency between the anti-inflammatory *Bifidobacterium* and the pro-inflammatory *Clostridia* and *Desulfovibrio* was reported*.* The growth of *Clostridia* is inhibited by lysozymes and by a higher concentration of *Bifidobacterium*. The authors proved that the increased proliferation of *Clostridia* in the gut, with a low concentration of *Bifidobacterium*, is a major risk factor for the development of leaky gut in patients with ASD [48]. This was ascertained by Adams et al. [49], who reported that lysozyme levels were significantly lower in autistic children (–27%, p = 0.04), possibly associated with probiotic usage. They hypothesized that probiotics provide limited defense against pathogenic bacteria, thus decreasing the need for the immune system to excrete lysozyme [49].

Calprotectin is a calcium- and zinc-binding protein that is usually released on the activation of neutrophilic granulocytes. The incidence of calprotectin in feces shows neutrophil infiltration into the intestinal tract and the level of fecal calprotectin (FC) is associated with macroscopic and histological inflammation. FC is stable in feces, and can be measured as a marker of intestinal inflammation–induced leaky gut [49, 50]. While Fernell et al. [51] reported the absence of an association between FC as a marker of intestinal inflammation and autism, Iovene et al. [42] found a significant positive correlation between Childhood Autism Rating Scale as measure of autism severity, abundance of *Clostridium spp.,* and calprotectin value. This suggests that gut inflammation could also participate in gut discomfort, finally leading to more severe behavior. These discrepancies can be attributed to the differences in the studied populations, the sensitivity of the measured technique, and the scores used to measure autistic severity. This suggestion was supported by the findings of a recent study by Babinská et al. [52], who found significant correlations between FC and all areas of the Autism Diagnostic Interview-Revised (ADI-R) diagnostic tool measured as impaired social interaction and communication, restrictive and repetitive behavior. This suggests that low-grade intestinal inflammation might be one of the major factors that involved in the etiology of ASD.

The permeability of the intestinal epithelium to small substances depends on the regulation of intercellular TJs. The zonula occludens (ZO-1) toxin (Zot, or zonulin) is an enterotoxin secreted by gut epithelial cells after stimuli from diets or microbiota. It is a potent regulator of TJ capability and intestinal barrier function. Notably, the interruption of the intestinal barrier in response to pathogenic microbial infection permits the passage of pro-inflammatory cytokines from the gut to the blood. Zonulin reduces the expression of intestinal TJ proteins, induces T-cell-mediated mucosal inflammation, and controls the transmigration of immune cells from the gut to the blood [53]. Zonulin has been shown to prompt the disassembly of TJs between the cells of the duodenum and the small intestine, resulting in leaky gut [53]. Multiple studies indicate that serum zonulin levels are associated with increased intestinal permeability, affecting neural, hormonal, and immunological pathways, and thus, could induce neurological abnormalities in the case of leaky gut [54]. Zonulin levels and Social Responsiveness Scale scores in patients with attention-deficit hyperactivity disorder (ADHD) differed significantly from those in controls. Elevated zonulin levels were associated with increased symptoms of hyperactivity and impairment of social functioning [55].

Another potential marker of gut integrity is intestinal fatty-acid-binding protein (I-FABP), also known as FABP2 [56]. This cytoplasmic FABP2 is found in the enterocytes of the small intestine, and elevated levels indicate cell damage [57, 58]. In some cases, it may be possible to assess the integrity of the epithelium through the measurement of intestinal I-FABP to monitor epithelium turnover in the jejunum, ileum, and colon [59].

Although zonulin and I-FABP have not been studied in neurological disorders, their principal mechanisms are presently unidentified, however, it has been hypothesized that more frequent gut epithelial cell death or dysfunction might decrease the expression of zonulin [60], suggesting that low plasma zonulin levels could be indicative of leaky gut. Soluble CD14 (sCD14) is a co-receptor for LPS, which is considered to be an activation marker for monocytes and other blood mononuclear cells released after stimulation [61]. LPS induce the secretion of sCD14 from immune cells [62]; hence, high plasma levels of sCD14 are thought to reflect exposure to LPS [63]. sCD14 is increased in conditions thought to be characterized by greater gut permeability, such as celiac disease [64], potentially as a consequence of bacterial translocation across the gut membrane [65].

Most recently, Vojdani et al. [66] postulated that the chemical modification of food proteins by diverse food toxicants might result in immune reactions that involve cross-reactions with tissue antigens, resulting in autoimmune reactivity. Leaky gut in patients with ASD, of course, can help toxins to cross the gut barrier, penetrating into different organs, where they can initiate autoimmune responses. Understanding the potential link between specific food consumption and autoimmunity in humans might set the foundation for additional research into the proper diet for the prevention of autoimmune diseases. Interestingly, calprotectin, zonulin, and lysozyme were found among the anti-egg antibody reactions that might explain the sensitivity of autistic patients to food. This might be the reason for the atypical eating behaviors observed in autistic children with leaky gut and autoimmunity. This might help to inform clinicians early enough so that they can identify autistic patients and develop evidence-based intervention strategies [66].

It is fascinating to know that in the patients with ASD studied, 75% of the ASD samples had downregulated gene expression of CLDN-1, OCLN, and TRIC, as barrier-forming TJ proteins, whereas 66% had upregulated expression of CLDN-2, -10, and -15 as pore-forming CLDNs compared to the controls [38]. It is concluded that in the ASD-affected brain, there is an altered expression of the genes associated with BBB integrity, coupled with increased neuroinflammation and possibly impaired gut barrier integrity [38]. There are great controversies regarding the role of pro-inflammatory cytokines in ASD. Despite the presence of a specific plasmatic cytokine profile in children with regressive ASD, recently, Prosperi et al. [67] did not support the use of cytokines as markers of leaky gut and did not support anti-inflammatory therapies in ASD-affected children. They recommended the search of alternative hypotheses for the etiology of GI symptoms in ASD-affected individuals. Based on their study, cytokines could not be used as markers of leaky gut in patients with ASD. The reason behind these discrepancies could be attributed to the heterogeneous nature between individuals with ASD. Subsets of autistic individuals with concomitant immunological illnesses, developmental deterioration, or high irritability might be more likely to benefit from anti-inflammatory therapies [68].

Leaky gut could also be related to the lower number of Lactobacilli in patients with ASD [42]. As mentioned earlier, this bacterium is usually involved in the maintenance of TJs in the intestinal epithelial barrier [69], and its diminution has been associated directly with chronic constipation in typically normal children [69]. As a result of the impairment of the intestinal barrier, the entry of toxins and bacterial metabolites into the bloodstream is permitted, and bacterial translocation into the mesenteric lymphoid tissue is favored, where they activate the immune system. It should be noted that although mucosal barrier impairment is the most studied mechanism connecting GI co-morbidity and intestinal dysbiosis in ASD, this relationship appears to be much more complex. In light of this, recent research suggests a relationship between the microbial profile in patients with ASD and an altered metabolism and absorption of disaccharides in their gut epithelium [34, 70].

Persico and Napolioni [71] recorded the elevation of urinary p-cresol and its conjugated derivative p-cresylsulfate in autistic patients compared to healthy controls. This elevation was associated with the female sex, clinical severity irrespective of sex, and behavioral regression as autistic phenotype. Higher p-cresol levels in patients with ASD were attributed to their altered gut microbiota, chronic constipation, antibiotics, and leaky gut [71]. Excessive p-cresol production and absorption could induce leaky gut either directly or through inflammatory mechanisms. An increased intestinal transit time due to chronic constipation was the most important factor behind the elevation of urinary p-cresol levels in young children with ASD [72]. Persico and Napolioni [71] reported the contribution of p-cresol in worsening autism severity and gut dysfunction. This might suggest p-cresol to be a member of a multi-biomarker diagnostic panel that can be used for the early diagnosis of autism in children.

In an attempt to correlate mitochondrial dysfunction as an etiology of autism with gut leakiness [73], induced toxicity was reported to increase the activity of Cytochrome P450-2E1 (CYP2E1) and inducible nitric oxide synthase (iNOS) [74], whereas toxicants suppress the mitochondrial electron transport chain, leading to the elevated production of reactive oxygen species (ROS) and reactive nitrogen species (RNS), resulting in increased oxidative/nitrative (nitroxidative) stress with production of potently toxic peroxynitrite that can nitrate Tyr residues and/or S-nitrosylate Cys residues. Based on elevated nitroxidative stress in ASD [75–77], it was hypothesized that many proteins, especially in intestinal epithelial junctional complexes, including TJ and adhesion junction proteins, are altered by post-translational modifications (PTMs) (e.g., oxidation, nitration, phosphorylation, adduct formation, etc.), followed by their proteasomal degradation and/or suppression, contributing to increased gut leakiness and serum endotoxin levels. Experiments utilizing GF mice indicated that the presence of healthy intestines with normal flora helps maintain the integrity of the BBB by positively regulating the expression of the TJ proteins occludin and claudin-5 [78].

**Fig. 2:**
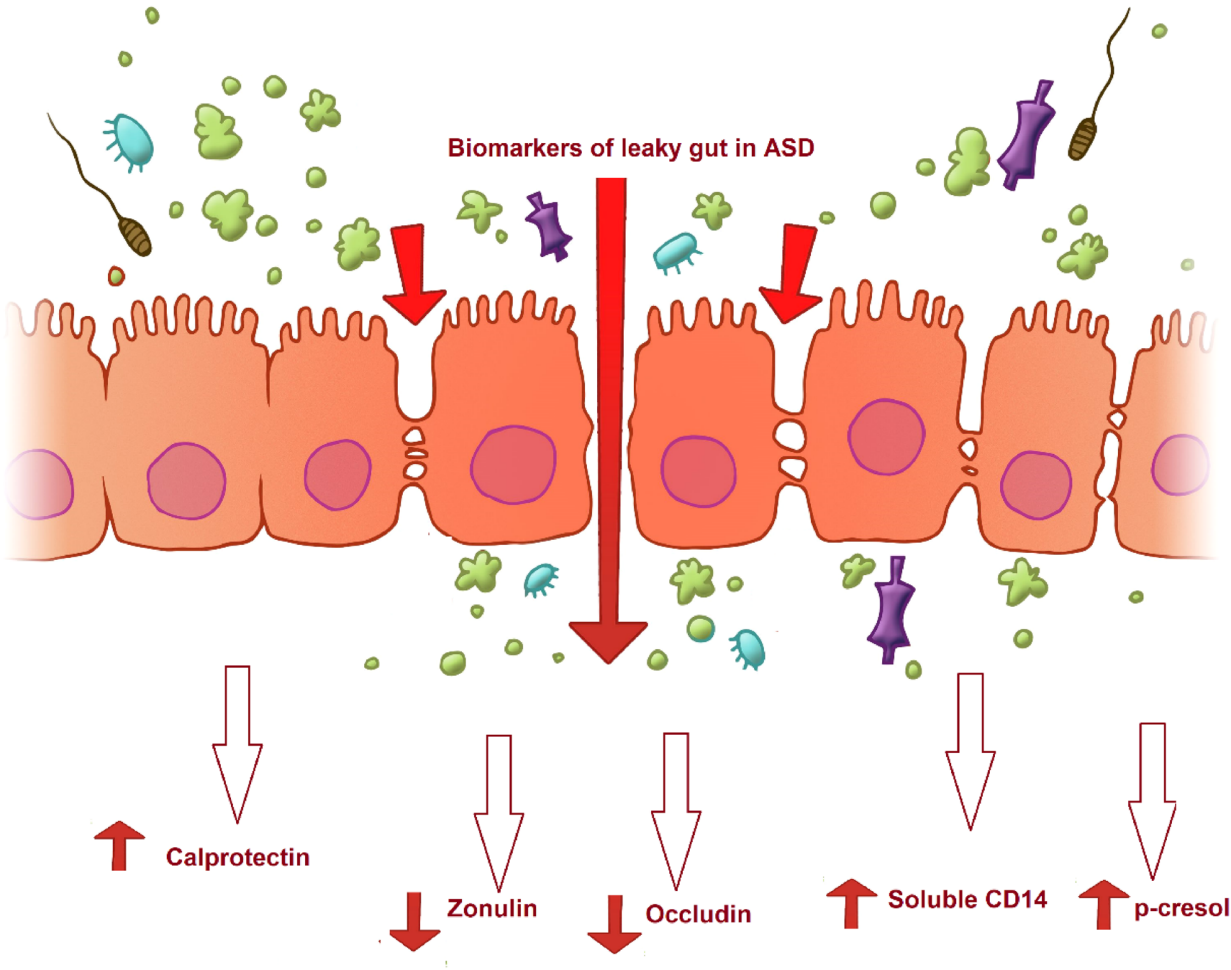
Relationship between leaky gut and ASD demonstrating the increase of calprotectin, soluble CD14 as co-receptor of LPS, and p-cresol as toxic bacterial metabolite together with the decrease of zonulin and occludin as predictive biomarkers of leaky gut in autistic patients

A phenylalanine metabolite [3-(3-hydroxyphenyl)-3-hydroxypropanoic acid], as biomarker of *Clostridia* spp. overgrowth, was remarkably elevated in the urine of patients with ASD and associated with ASD-like behaviors in rodent models [79] (Fig. 2).

## Leaky gut and brain neurotransmitters

In an attempt to understand the role of leaky gut in dysbiosed microbiota on synaptopathy or abnormal neurochemistry of the brain, it is interesting that gut bacteria seem to regulate several key neurotransmitters, such as gamma-amino butyric acid (GABA), glutamate, serotonin, and dopamine [80–82] that have shown altered levels in patients with ASD [83]. In fact, an imbalance in the CNS between excitation (glutamate) and inhibition (GABA) has been postulated to contribute to ASD [84–86]. In addition, bacteria can produce a wide range of neuro-endocrine hormones that can intervene in intestinal homeostasis and modulate mood and behavior [87, 88]

El-Ansary et al. [82] reported that male Syrian hamsters exposed to a neurotoxic dose of propionic acid show ASD-like behaviors and glutamate excitotoxicity in the brain. A probiotic mixture of *Bifidobacterium breve*, *Bifidobacterium infantis*, *Lactobacillus acidophilus*, *Lactobacillus bulgaricus*, *Lactobacillus casei*, *Lactobacillus rhamnosus*, and *Streptococcus thermophiles* reduced the ASD effects induced by high-dose propionic acid. The deletion of the *SHANK3* gene is associated with dysregulated neurodevelopment and autistic-like behaviors [86]. In Shank3 knockout mice, there was a decrease in the level of *Lactobacillus reuteri* colonization, which correlated with the decreased expression of GABA receptor subunits in the brain; *L. reuteri* supplementation attenuated unsocial behavior in male Shank3 knockout mice and increased GABA receptor expression [89].

It is fascinating to know that elevations of blood 5-hydroxytryptamine (5-HT) concentrations in individuals with ASD may be indicative of dysregulation of GI 5-HT secretion. Actually, elevated whole-blood 5-HT levels have been associated with constipation in patients with ASD [90]. On the other hand, increasing evidence supports the idea that specific intestinal bacteria may play significant roles in regulating levels of gut cell-derived 5-HT and whole-blood 5-HT [90]. This may be a direct result of impaired bacterial production or secondary to the effects of intestinal metabolites produced by the microbiota. For example, SCFAs, which are produced by enteric bacteria that ferment dietary saccharides(including Clostridium species), have been shown to increase levels of Tph1mRNA in EC cells and, subsequently, increase intestinal 5-HT levels without changing SERT expression [90]. Further, fecal metabolites produced by spore-forming bacteria, particularly Clostridium species, have been shown to increase 5-HT levels in EC cell cultures, as well as in the colon and blood of GF mice [43]. More definitely, it has been hypothesized that polymorphism in the gene that encodes the serotonin transporter (SLC6A4) was associated with the increase in serotonin-producing microbes, such as *Candida, Streptococcus, Escherichia, Enterococcus*, and *Clostridium* in patients with ASD. This could increase the intestinal production of serotonin at the expense of a lower synthesis in the brain, because of the consumption tryptophan as serotonin precursor, leading to hyperserotoninemia and intestinal dysmotility as autistic features [79, 90, 91].

Furthermore, the Gram-positive intestinal bacteria *Lactobacillus* sp. And *Bifidobacterium* sp. Can metabolize glutamate to produce GABA, which is a major inhibitory neurotransmitter of the CNS, known to be lower in patients with ASD and associated with sensory hyper-responsiveness [92].

## Role of diet in leaky gut

**Fig. 3:**
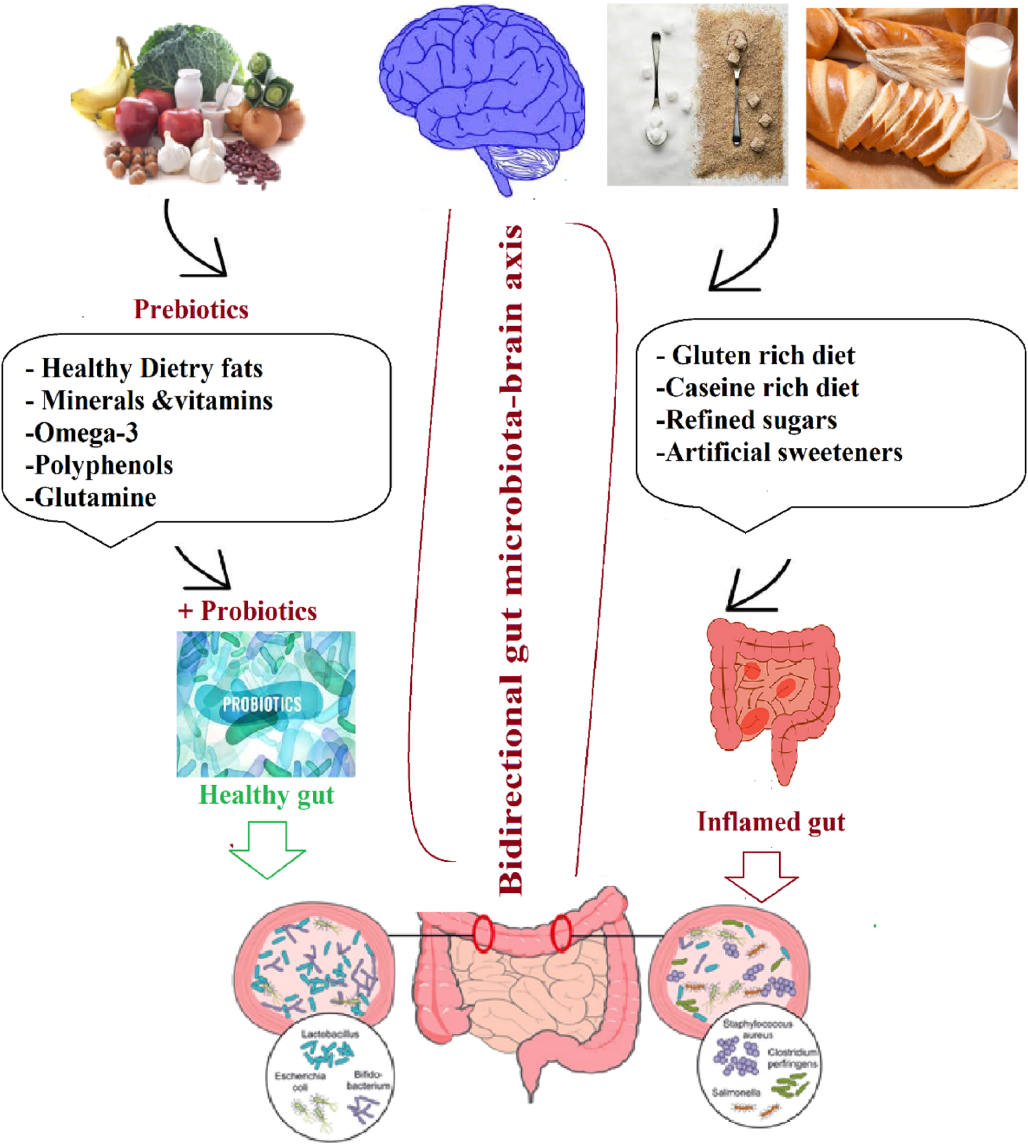
Interaction between diet, gut microbiota, and the brain. Healthy prebiotic food usually accompanied with healthy gut with higher growth of probiotics, while food such as gluten/ casein-rich, refined sugars, artificial sweeteners and others show alteration of the gut microbiota composition, leading to inflamed or leaky gut with subsequent changes in the behavior profile through the microbiota-gut-brain axis

Healthy gut microbes play a significant role in retaining the integrity of the gut lining. Some SCFAs and proteins that are mainly responsible for strengthening the intestinal wall are produced by healthy gut flora in the body. The loosening of gaps present in the intestinal wall because of the presence of unhealthy gut microbes could result in leaky gut. Microbial communities in the gut are mainly modulated by diet, as any modification in the diet regime has a direct impact on the composition and function of the gut microbiota [93]. A small change in the diet can quickly change the gut flora, which in turn can affect the physical and mental well-being of a person. Gut flora have a complex metabolic capacity. Some bacterial species in the gut compete with each other for the same kind of food. As a result, species that compete less get lower abundance. For this reason, the notion of introducing prebiotic compounds in the diet to maintain human gut health is proposed everywhere these days [94, 95]. Currently, the concept of using a specific kind of dietary fiber in food to introduce the growth of desired gut microbes is also recommended [95]. In almost all countries, dietary fiber is considered a healthy diet, which is present mainly in fruit, vegetables, and cereals [96]. Dietary fiber is rich in complex carbohydrate molecules called glycans, which are not digested by human enzymes. Most often, these fibers are either metabolized by gut microbes or excreted undigested in the feces. Supplements such as prebiotics and probiotics that are capable of modulating the gut microbiota can prove helpful in the prevention leaky gut, whereas some foods can damage the lining of the gut, causing leaky gut. The associations between leaky gut and different kinds of food are listed below (Fig. 3).

### Diet that prevents leaky gut

#### Prebiotics

Prebiotics stimulate the growth of a limited number of taxa in the gut, which confers health benefits [97]. Prebiotic dietary fibers act as a carbon source for the growth of beneficial host microbes, as most of them are oligosaccharides [98]. Prebiotic dietary fibers can produce beneficial metabolites, increase calcium absorption, reduce protein fermentation, enhance immune system defense, reduce pathogenic bacteria populations, and help in maintaining gut barrier permeability. Prebiotics are present in diverse food groups. The most common prebiotic consumed by humans in the natural form is inulin, as it is present in many naturally occurring fruits and vegetables [98]. Inulin-containing foods can significantly increase the growth of *Bifidobacteria*, which is actually a probiotic that is beneficial to the host’s health [99]. Other prebiotics that are vital for gut health are fructooligosaccharides and galactooligosaccharides. Both are oligosaccharides that occur in many plants in their natural form. Healthy bacteria, such as Actinobacteria, Bacteroidetes, and Firmicutes, can digest both fructooligosaccharides and galactooligosaccharides in the gut. The digestion of prebiotics by gut bacteria maintains its pH, as fermentation products of prebiotics are mostly acids [100, 101]. The acidic environment can change the population of acid-sensitive species like Bacteroides, and can stimulate butyrate formation by Firmicutes, collectively called the butyrogenic effect [101].

#### Probiotics

Probiotics, as defined by the Food and Agriculture Organization/World Health Organization, are live microorganisms that when given in adequate amounts confer benefits to the host. The probiotic microorganisms present most frequently in the diet and used as probiotic supplements include *Lactobacillus* sp., among which are *L. acidophilus*,L. *brevis; L. casei, and L. plantarum* bacterial strains in addition to *Bifidobacterium* sp., among which are *B. infantis, B. adolescentis, B. animalis*, *Streptococcus* sp., and *Enterococcus* sp., which are lactic-acid-producing bacteria; and *Bacillus* and *Propionibacterium*, which are non-lactic-acid-producing bacteria. Also, some nonpathogenic yeast-like *Saccharomyces* and *Coccobacilli* are included as probiotics [102]. Probiotics are present usually in yogurt, cultured buttermilk, and cheese. Probiotic bacteria can survive in conditions of low pH, which are provided by these dairy products. Some nondairy fermented grains and vegetables, such as maize, pearl millet, cereals, legumes, and cabbage, can also enhance the growth of these strains [103]. Yogurt was the first food where probiotics were added; however, nowadays, new probiotic food, such as ice cream, chocolate, beverages, cereals, and vegetable products, are also available in the market [104]. Also, some probiotic supplements are available in the market in the form of pills, capsules, tablets, syrups, and powders. Probiotics have been shown to be effective against GI disorders such as leaky gut. Probiotics maintain normal mucosal homeostasis, protect against mucosal injury, and strengthen epithelial TJs [105]. Bioactive factors released by probiotics can trigger the activation of various cell signaling pathways that can decrease leaky gut by enhancing mucosal integrity. Some probiotics are capable of enhancing the upregulation of junction-associated proteins such as ZO-1. Increased ZO-1 expression results in reduced intestinal permeability through the enhancement of junctional complexes [105].

#### Gut-healing supplements

Zinc: Zinc supplementation improves intestinal barrier function and intestinal permeability [106]. Zinc maintains the mucosal barriers by enhancing the expression of occludin and ZO-1 proteins, resulting in the tightening of junctions [107, 108]. It can even protect against intestinal dysfunction and intestinal leakage induced by bacterial toxins [109]. Red meat and shellfish, such as oysters, crab, mussels, and shrimp, are excellent sources of zinc. Vegetarian sources include nuts, beans, and whole grains [110].

Glutamine: Glutamine is one of the best supplements used to treat leaky gut, as it acts as a fuel for enterocytes and colonocytes [111]. Moreover, it maintains gut barrier function and prevents permeability to toxins and pathogens under various conditions of GI mucosal injury. A low serum glutamine concentration correlates with intestinal barrier disruption. Dietary supplementation in glutamine maintains gut mucosa during sepsis, infection, radiation, and various other catabolic stress conditions. It can also regulate mucosal epithelial TJ integrity. In the gut physiology, glutamine promotes enterocyte proliferation, regulates TJ proteins, suppresses pro-inflammatory signaling pathways, and protects cells against apoptosis and cellular stresses. It is the most abundant free amino acid present in the human body and is a major substrate utilized by intestinal cells [112]. The human gut has little capacity to synthesize glutamine, so it relies mainly on the diet for its supply [113]. Foods rich in glutamine include beef, chicken, fish, dairy products, eggs; vegetables such as beans, beets, cabbage, spinach, carrots, parsley, brussels sprouts, celery, kale, and vegetable juices, as well as wheat and pawpaw [114].

Healthy dietary fat: Avocado oil, coconut oil, and extra virgin olive oil have anti‐inflammatory properties, and their consumption could be favorable for the host microbial ecosystem. These oils increase the populations of *Bacteroidetes* [115]. Similarly, an omega-3-rich high-fat flaxseed/fish oil diet leads to the increased presence of *Bifidobacterium* sp. in the gut. Nuts such as peanuts, almonds, and walnuts are also considered good for the gut and prevent leaky gut. The increased consumption of omega-3 fatty acids found in fatty fish leads to increased circulating docosahexaenoic acid levels, which correlates with high levels of *Lachnospiraceae* and *Ruminococcacae* families, which are involved in the fermentation of dietary fibers. In addition, the dietary intake of polyphenols, vitamins, and other micronutrients has the capacity to shape the gut microbiome [115].

Dietary polyphenols: Foods rich in polyphenols, such as catechins, flavonols, flavones, anthocyanins, proanthocyanidins, and phenolic acids, include fruits, seeds, vegetables, tea, and cocoa [116]. These foods are enriched with *Bifidobacterium* and *Lactobacillus*, and are vital in preventing IBD [116, 117]. They also reduce pathogenic *Clostridium* species (*C. perfringens* and *C. histolyticum*) in the gut [118, 119].

### Avoidance of leaky-gut-promoting diets

#### Gluten-rich diet

Gluten is a complex molecule consisting of gliadin and glutenins, which are found in wheat, rye, and barley. These proteins are mostly insoluble and difficult to digest because of their high content of proline and glutamine. These proteins can induce changes in the gut permeability and loss of gut TJ barrier function, resulting in leaky gut [120]. Numerous studies have confirmed that gliadin can increase the intestinal permeability via zonulin [120, 121]. Gluten can act as a food antigen in blood, where it can trigger inflammation. Sometimes, an antibody to gluten can have cross-reactivity with casein (milk protein), resulting in a similar immune reaction. A gluten-free diet is always recommended for people suffering from gut diseases because the ingestion of gluten causes the destruction of the intestinal villi in these patients [122, 123].

#### Refined sugars

Refined sugars, such as table sugar, sodas, pastries, bread, pasta, etc., act as the main food sources for many pathogenic microbes present in the intestines. By consuming too much refined sugars, the populations of health-promoting bacteria in the digestive system are reduced, and the populations of pathogenic bacteria and fungi like *Candida albicans* flourished, which can result in leaky gut. As a result of the increased proliferation of pathogenic bacteria, exotoxins cause gut tissue inflammation and undigested proteins are passed to the lumen. Undigested proteins act as antigens that are detected by gut-associated lymphoid tissue (GALT), resulting in inflammation [122]. Due to inflammation, villi on the enterocytes are destroyed, and enzymes produced by those villi, such as lactase, sucrase, and maltase, disappear, resulting in the decline of the digestion process of carbohydrates, which are then flushed down to the lower intestine, where they can feed pathogenic bacteria that cause leaky gut symptoms by fermentation [122].

#### Artificial sweeteners

Artificial sweeteners can cause microbiome changes in the gut, leading to inflammation and leaky gut [123]. Artificial sweeteners mimic the sweet taste without providing energy. Sweeteners can alter glucose metabolism and increase appetite stimulation [124]. The consumption of some sweeteners, such as saccharin, sucralose, and aspartame, causes alterations in the gut microbiota and in blood glucose levels [125, 126].

## Conclusion

In this article, we review evidence from numerous studies presenting the association between leaky gut and altered gut microbiota in patients with ASD. We review the most important biomarkers of leaky gut as early diagnostic tools in ASD. Finally, we describe some possible food intervention strategies to modulate the gut microbiota in individuals with ASD. Many recent clinical studies have demonstrated that actions that normalize the gut microbiota cause remarkable improvements in ASD symptoms. Nevertheless, well-designed clinical trials with large numbers of participants are recommended.

## Challenges and limitations

Regardless of the growing evidence that endogenous markers are involved in the pathology of ASD, early identification of leaky gut-associated biomarkers remains a major challenge**.** Animal models used for the study of the gut microbiota-ASD relationship are limited and are mostly inbred or environmentally-induced models which is less valid than genetic models. Moreover, the heterogeneous nature between individuals with ASD is greatly contributed in many reported decripences between and among studies investigating contribution of gut microbiota alteration in ASD, and the use of probiotics as anti-inflammatory therapies that may treat gut leakiness. There is only few clinical and well-designed studies that include enough number of patients, to provide strong evidence that supports the use of probiotics, dietary and supplement as treatment strategies.

English Editing certificate showing the revision of the manuscript by Enago the editing brand of Crimson Interactive Inc. was provided by the corresponding author in response to editorial comment (Additional file 1).

## Supplementary Information

**Additional file 1: Figure S1.** English editing certificate.

## Data Availability

Not applicable.

## Abbreviations

ADHD: Attention deficit hyperactivity disorder
AJ: Adhesion junction
ASD: Autism spectrum disorder
BBB: Blood brain barrier
CARS: Childhood Autism Rating Scale
CCL2: Inflammatory cytokines chemokine
CD14: Cluster of differentiation 14
CNS: Central nervous system
CRP: C-reactive protein
CYP2E1: Cytochrome P450-2E1
DHA: Docosahexaenoic acid
GF: Germ-free mice
GABA: Gamma amino butyric acid
GI tract: Gastrointestinal tract
GALT: Gut-associated lymphoid tissue
HBI: Harvey-Bradshaw Index
5HT: 5-Hydroxytryptamine
IBD: Inflammatory bowel disease
IEC: Intestinal epithelial cells
I-FABP: Intestinal fatty acid binding protein
IFN: Immune interferon
iNOS: Inducible nitric oxide synthase
LPS: Lipopolysaccharide
PTMs: Post-translational modifications
RNS: Reactive nitrogen species
ROS: Reactive oxygen species
SCFA: Short chain fatty acid
SLC6A4: Serotonin transporter
SRS: Social responsiveness scores
TJ: Tight junction
ZO: Zonula occludens
