# Supplementary material for: The use of biomarkers associated with leaky gut as a diagnostic tool for early intervention in autism spectrum disorder: a systematic review
Source: Gut Pathog. 2021 Sep 13;13:54. doi: 10.1186/s13099-021-00448-y (PMC8439029; doi:10.1186/s13099-021-00448-y)
Supplement: Supplementary file 1 — Additional file 1: Figure S1. English editing certificate. [file 13099_2021_448_MOESM1_ESM.pdf]

enago — Author First, Quality First — www.enago.com | ulatus — localize to globalize — www.ulatus.com | voxtab — www.voxtab.com

# CERTIFICATE OF EDITING

This is to certify that the paper titled **Ramesa Shafi Bhat  The use of biomarkers associated with leaky gut as a diagnostic tool for early intervention in autism spectrum disorder: A systematic review** commissioned to us by **Ramesa Shafi Bhat** has been edited for English language, grammar, punctuation, and spelling by Enago, the editing brand of Crimson Interactive Inc. under Copyediting.

✔ **ISO 17100:2015**
Translation Service Providers

✔ **ISO 27001:2013**
Information Security Management System

✔ **ISO 9001:2015**
Quality Management System


**Issued by:**
**Enago, Crimson Interactive Inc.**
**160, Greentree Dr, Ste 101 street,**
**Dover City, Kent, Delaware, 19904**
**Phone: +1-302-498-8358**


**Disclaimer :** The intent of the author's message has been preserved during the editing process. The author is free to accept or reject our changes in the document after reviewing our edits. This certificate has been awarded at the time of sharing the final edited version (full file or sections of the file) with the author. Enago does not bear any responsibility for any alterations done by the author to the edited document post 17 Mar 2021.

---

**Japan** www.enago.jp, www.ulatus.jp, www.voxtab.jp
**Taiwan** www.enago.tw, www.ulatus.tw
**China** www.enago.cn, www.ulatus.cn
**Brazil** www.enago.com.br, www.ulatus.com.br
**Germany** www.enago.de

**Russia** www.enago.ru
**Arabic** www.enago.ae
**Turkey** www.enago.com.tr
**S. Korea** www.enago.co.kr
**Global** www.enago.com, www.ulatus.com, www.voxtab.com

**About Crimson:**
Crimson Interactive INC is one of the world's leading academic research support services. Since 2005, we've supported over 2 million researchers in 125 countries with their publication goals.
